# Factors Associated With Depressive and Anxiety Symptoms, Barriers, and Facilitators for Seeking Support Among European University Students: A Cross‐Sectional Multicenter Study

**DOI:** 10.1155/da/8994187

**Published:** 2026-04-10

**Authors:** Lara Guedes Pinho, Anabela Afonso, Lisa Ryan, Ed Daly, Ana Morais, Maria Engström, Annika Nilsson, Javier Cubero, Christophe Jarry, Mariana Muresan, Pedro Amaro, Tomina Saveanu, Maria Revés Silva, Roberto Sala, Giuseppe Marletta, César Fonseca, Adriana Borza, Gonçalo Jacinto

**Affiliations:** ^1^ Comprehensive Health Research Centre (CHRC), LA_REAL, Universidade de Évora, Évora, Portugal, uevora.pt; ^2^ Escola Superior de Saúde, Instituto Politécnico de Viana do Castelo, Viana do Castelo, Portugal, ipleiria.pt; ^3^ Centro de Investigação em Matemática e Aplicações (CIMA), Universidade de Évora, Évora, Portugal, uevora.pt; ^4^ Escola de Ciências e Tecnologia, Universidade de Évora, Évora, Portugal, uevora.pt; ^5^ Dept of Sport, Exercise and Nutrition, Atlantic Technological University, Galway, Ireland; ^6^ Escola de Saúde e Desenvolvimento Humano, Universidade de Évora, Évora, Portugal, uevora.pt; ^7^ Department of Caring Science, Faculty of Health and Occupational Studies, University of Gävle, Gävle, Sweden, hig.se; ^8^ Faculty of Education and Psychology, Department of Science and Mathematics Teaching, Health Education Lab, University of Extremadura, Badajoz, Spain, unex.es; ^9^ Laboratoire de Psychologie des Pays de la Loire, LPPL EA 4638, SFR Confluences, UNIV Angers, Nantes Université, Angers, France, laris.univ-angers.fr; ^10^ Service de Santé, Universitaire d’Angers, Angers, France, lunam.fr; ^11^ Department of Preclinical Disciplines, Medicine and Pharmacy Faculty, University of Oradea, Oradea, Romania, uoradea.ro; ^12^ CARE – Research Center on Health and Social Sciences, Portalegre Polytechnic University, Portalegre, Portugal; ^13^ Management-Marketing Department, Faculty of Economic Sciences, University of Oradea, Oradea, Romania, uoradea.ro; ^14^ Escola Superior de Enfermagem São João de Deus, Universidade de Évora, Évora, Portugal, uevora.pt; ^15^ Dipartimento di Medicina e Chirurgia, Università degli Studi di Parma, Parma, Italy, unipr.it; ^16^ Azienda Ospedaliero-Universitaria Parma, Parma, Italy, ao.pr.it; ^17^ Department of Career and Psychological Counseling, and Department of Psychology, University of Oradea, Oradea, Romania, uoradea.ro; ^18^ Faculdade de Ciências e Tecnologia, Universidade do Algarve, Faro, Portugal, ualg.pt; ^19^ Center for Research and Development in Mathematics and Applications (CIDMA), Department of Mathematics, University of Aveiro, Aveiro, Portugal, ua.pt

**Keywords:** anxiety disorders, depressive disorder, health services accessibility, help-seeking behavior, students

## Abstract

**Aim:**

The rationale for this current study was to assess the factors associated with anxiety and depressive symptoms in a cohort of students from seven universities across the European education system. Furthermore, the research sought to assess the barriers and facilitators for seeking support when students perceive any mental health issues.

**Methods:**

A cross‐sectional multicenter study was conducted, which included 4830 higher education students from seven European countries. A sociodemographic questionnaire, the Patient Health Questionnaire (PHQ‐9), and the Generalized Anxiety Disorder (GAD‐7) were used to assess depressive and anxiety symptoms, respectively. The Mental Health Inventory (MHI‐5) assessed general psychological well‐being. A questionnaire was used to assess preferences regarding support‐seeking behavior and perceived barriers in the event of mental distress. Linear mixed models (LMMs) were used in the statistical analysis.

**Results:**

Swedish students reported lower levels of moderate to severe depressive symptoms (34.1%) and moderate to severe anxiety (27.9%) when compared, for example, with Irish students (63.1% and 52.9%, respectively). Younger students, females, students with a history of mental disorder and lower levels of academic performance, or from a poorer socioeconomic background reported increased rates of depressive and anxiety symptoms. The most valued support‐seeking strategies were speaking to friends or engaging in psychotherapy; in contrast, the most cited barrier to seeking support was the expense related to professional therapy and long waiting times for an appointment with professional therapists.

**Conclusion:**

There is a need to develop and/or review mental health promotion strategies for higher education students across Europe. These strategies need to consider the individual and culturally specific needs of higher education students so that they are effective in removing perceived barriers when seeking support in the event of mental distress.

## 1. Introduction

University students face many challenges that can impact their mental health, beyond the expected stressors associated with higher education [1]. The transition to university represents a critical period of personal and academic growth, yet it also introduces demands such as academic pressure, financial concerns, social adjustment, and uncertainties regarding future employment [2, 3]. Depression and anxiety are increasingly prevalent in this population, posing significant risks to academic performance, social development, and overall well‐being [4–6].

The development of mental disorders during university is influenced by a combination of individual, social, and environmental factors. Academic demands [7], social isolation, and lack of support networks [8], financial pressures [9], and, for international students, cultural adaptation or, in some cases, discrimination [10]. Therefore, it is important that in academic environments the staff pay attention to all these factors and to the coping strategies used by students.

Untreated mental health issues are associated with disengagement from learning, underperformance, withdrawal from programs, and elevated risks of substance abuse, eating disorders, and suicidal ideation [11, 12]. Coping mechanisms such as substance use may further amplify mental health difficulties [13, 14].

Universities play a central role in mitigating these challenges through the provision of counseling, psychotherapy, peer‐led programs, and digital interventions [15–17]. Evidence‐based approaches, including cognitive–behavioral therapy and mindfulness‐based stress reduction, have been shown to reduce depressive and anxious symptoms [18]. Mental health literacy campaigns and antistigma initiatives further support student well‐being [19, 20]. However, barriers such as limited resources, long waiting times, cultural stigma, and lack of awareness of available services can limit the effectiveness of these strategies [21–23].

Despite growing recognition of these challenges, gaps remain in understanding the specific factors associated with anxiety and depression across different European university contexts, as well as the barriers and facilitators influencing help‐seeking behaviors. Guided by a socioecological framework of mental health, which considers the interplay between individual, social, and institutional factors, this study aimed (1) to assess the factors associated with anxiety and depressive symptoms in a cohort of students from seven universities across the European education system and (2) to assess the barriers and facilitators for seeking support when students perceive any mental health issues. By addressing these gaps, the study provides insights to inform the needs of higher education students that may be useful for the future implementation of strategies to promote mental health or intervene when a mental disorder already exists.

## 2. Methods

### 2.1. Study Design

This study used a descriptive, comparative, and cross‐sectional survey design and took place in seven universities, each from a different country (Portugal, Sweden, France, Ireland, Italy, Romania, and Spain). These seven universities are part of EU GREEN, which is a European university alliance focused on inclusive education and sustainability practices utilizing universities across Europe. This article is in line with STROBE Statement Guidelines for cross‐sectional studies.

This research project focused on three areas: (1) mental health (this study), (2) sexual and moral harassment, and (3) substance abuse, such as alcohol, tobacco, and medication. The last two will be the subject of another manuscript.

### 2.2. Population and Participant Selection

The target population for this study were university students attending one of the seven partner institutions. An email was sent to the students at the selected universities, inviting them to participate in this study. The inclusion criterion for the sample was being a university student at one of the universities and having access to the Internet. Only students who completed the authorized consent form were recruited to the convenience sample.

### 2.3. Data Collection

Data collection took place during the 2023/2024 academic year, starting in October 2023 and ending in July 2024. The questionnaires were applied online using the Google Forms application. The questionnaire link was disseminated via email to all students at each of the seven universities. When possible, students were asked to complete the questionnaire in class.

#### 2.3.1. Measurement Instruments

A sociodemographic characterization questionnaire was used with questions about background characteristics such as age, sex, nationality, emotional relationship, sexual orientation, socioeconomic level, residence, academic situation, perceived mental health status, types of barriers to ask for support, experience of moral and sexual harassment, and consumption habits.

The Mental Health Inventory (MHI‐5), a reduced version of the 38‐item MHI [24], consists of five items that assess psychological well‐being (two items) and the absence of psychological distress (three inverse‐scored items) in the last month. The answers, on a Likert scale, range from zero (none of the time) to five (all the time). Most samples showed a good internal consistency in MHI‐5: Ireland (0.86), Italy (0.79), Portugal (0.87), Romania (0.84), Spain (0.82), and Sweden (0.88), but France showed an unacceptable internal consistency (*α* = 0.43).

Using the Generalized Anxiety Disorder (GAD‐7) assessment scale to measure anxiety symptoms over the past 2 weeks [25]. The GAD‐7 consists of seven items, answered on a 4‐point Likert scale, from zero (not at all) to three (nearly every day). The maximum score is 21 points on each subscale, with higher scores showing a greater presence of anxiety symptoms. The cut‐off points are 0–4 (minimal), 5–9 (mild), 10–14 (moderate), and ≥15 (severe). Most samples showed excellent internal consistency in GAD‐7: France (0.87), Ireland (0.88), Italy (0.91), Portugal (0.91), Romania (0.92), Spain (0.91), and Sweden (0.90).

Using the Patient Health Questionnaire (PHQ‐9) to assess the frequency and severity of depression symptoms over the past 2 weeks [26]. The PHQ‐9 consists of nine items answered on a Likert scale, from zero (not at all) to three (nearly every day). The total scores range from 0 to 27, with higher scores associated with more severe depression. The cut‐off points are minimal (0–4), mild (5–9), moderate (10–14), moderately severe (15–19), or severe (20–27) depression. Good to excellent internal consistency was observed in PHQ‐9: France (0.86), Ireland (0.88), Italy (0.90), Portugal (0.90), Romania (0.90), Spain (0.91), and Sweden (0.88).

To assess the types of support most valued by students in the event of mental health concerns, a questionnaire was developed which included questions such as “Talking with friends,” “Medication,” or “Online intervention.” In reaction to the perception of barriers, items such as “Uncertainty regarding where to find help,” “I can’t imagine talking with a ‘stranger’,” or “Concerns about possible side effects of medications” were asked.

### 2.4. Statistical Analysis

Kolmogorov–Smirnov test with Lilliefors correction, Shapiro–Wilk’s test, graphical inspection of the histogram and *Q–Q* plot, and skewness and kurtosis coefficients were used to check the normality assumption. Levene’s test was used to evaluate heteroscedasticity.

#### 2.4.1. Forms of Help

Because not all countries used the same number of ordinal categories in the responses to forms of support, the responses were recategorized into four‐ordinal categories: unlikely (very unlikely + Unlikely), somewhat unlikely (somewhat unlikely + rather unlikely), somewhat likely (rather likely + somewhat likely), and likely (likely + very likely). All unsure answers from Portugal were classified as somewhat likely, since according to the language translation, it was the most accurate option. Unsure responses from Romania and Spain were randomly and equally divided into the somewhat unlikely (≅50%) and somewhat likely (≅50%) categories.

#### 2.4.2. Barriers

Not all countries used the same number of ordinal categories in responses to barriers to seek support. Ireland and Italy used six‐ordinal categories, while all other countries used five‐ordinal categories. Therefore, for Ireland and Italy, the category “very unimportant” was included with the “unimportant” category.

To check whether there was a significant difference between countries by type of support, the Kruskal–Wallis test was applied, followed by the Dunn test with Holm correction. The eta‐squared (η^2^) effect size statistic for the Kruskal–Wallis test was computed. To check whether there was a significant difference between the presence and absence of anxiety and depression symptoms, the Mann–Whitney Wilcoxon test was applied. The same approach was used to check if there were differences between countries by the barriers to seek support.

#### 2.4.3. *MHI-5*, *GAD-7, and PHQ-9 Scores*

To compare MHI‐5, PHQ‐9, and GAD‐7 scores by country, an analysis of variance was used; when required, the variables were transformed using the Box–Cox transformation. Welch’s correction was used if the assumption of homogeneity of variances was violated, while the Kruskal–Wallis test was used when there was a large departure from the normality of the residuals. In the post hoc analysis, the Games–Howell was used when parametric ANOVA was applied or the Dunn test when Kruskal–Wallis was used.

#### 2.4.4. Factors Associated With GAD‐7 and PHQ‐9 Scores

To identify the factors associated with higher PHQ‐9 and GAD‐7 scale values, a linear mixed model (LMM) [27] was used (estimated using REML and the Nelder–Mead simplex algorithm optimizer) to predict PHQ‐9 and GAD‐7 scores, using the following fixed effects: age, sex, emotional relationship, category of socioeconomic level perceived by the student, study cycle, year of study (1st vs. other), academic performance perceived by the student, being a student who was employed, presence of previous mental diagnosis, and taking psychiatric medication. Sex was dichotomized to female vs. male/other due to the lack of observations in the other category (Table 1). These potential confounders were selected based on prior literature. The model included countries as random effects to account for variability across countries.

**Table 1:** Sociodemographic characteristics of the sample by country (*n* and valid %).

Variables	France (*n* = 198) *n* (%)	Ireland (*n* = 514) *n* (%)		Italy (*n* = 1226) *n* (%)	Portugal (*n* = 1135) *n* (%)	Romania (*n* = 509) *n* (%)	Spain (*n* = 632) *n* (%)	Sweden (*n* = 616) *n* (%)
Sex								
Female	154 (79.4)		368 (71.6)	882 (72.1)	762 (67.3)	411 (80.7)	464 (73.4)	468 (76.0)
Male	29 (14.9)		146 (28.4)	342 (27.9)	371 (32.7)	98 (19.3)	167 (26.4)	141 (22.9)
Other	11 (5.7)		—	—	—	—	1 (0.2)	7 (1.1)
Emotional relationship								
Single	119 (61.7)		311 (60.5)	571 (46.7)	586 (52.0)	243 (47.7)	342 (54.4)	184 (29.9)
In a relationship, living together	31 (16.1)		78 (15.2)	141 (11.5)	410 (36.4)	101 (19.8)	53 (8.4)	344 (55.8)
In a relationship, not living together	43 (22.3)		125 (24.3)	511 (41.8)	122 (10.8)	162 (31.8)	232 (37.0)	71 (11.5)
Other	0		0	0	9 (0.8)	3 (0.6)	1 (0.2)	17 (2.8)
Sexual orientation								
Heterosexual	127 (65.8)		358 (69.9)	1042 (85.3)	913 (80.7)	458 (90.7)	486 (76.9)	—
Bisexual	35 (18.1)		91 (17.8)	113 (9.2)	108 (9.5)	15 (3.0)	96 (15.2)	—
Homosexual	14 (7.3)		27 (5.3)	57 (4.7)	41 (3.6)	10 (2.0)	31 (4.9)	—
Asexual	7 (3.6)		23 (4.5)	10 (0.8)	16 (1.4)	6 (1.2)	7 (1.1)	—
Other	—		13 (2.5)	0	19 (1.7)	1 (0.2)	1 (0.2)	—
Prefer not answer	10 (5.2)		0	0	34 (3.0)	15 (3.0)	11 (1.7)	—
Nationality								
From the country	186 (94.9)		408 (79.4)	1185 (96.7)	1044 (92.1)	490 (96.6)	607 (96.7)	550 (89.9)
From the country and other	—		1 (0.2)	1 (0.1)	1 (0.1)	0	1 (0.2)	8 (1.3)
Other	10 (5.1)		105 (20.4)	40 (3.3)	89 (7.8)	17 (3.4)	20 (3.2)	54 (8.8)
Socioeconomic level								
Low	26 (13.3)		80 (15.6)	77 (7.3)	78 (6.9)	20 (3.9)	41 (6.5)	86 (14.0)
Medium	151 (77.4)		403 (78.4)	800 (75.5)	958 (84.5)	375 (73.7)	528 (83.8)	468 (76.2)
High	18 (9.2)		31 (6.0)	183 (17.3)	98 (8.6)	114 (22.4)	61 (9.7)	60 (9.8)
Relocated from home								
No	49 (24.7)		306 (59.5)	753 (61.6)	244 (21.5)	238 (46.8)	248 (39.2)	516 (83.8)
Yes	149 (75.3)		208 (40.5)	470 (38.4)	890 (78.5)	271 (53.2)	384 (60.8)	100 (16.2)
If relocated, going home frequency								
Every weekend	28 (15.1)		108 (21.0)	72 (5.9)	356 (31.7)	62 (12.2)	161 (25.5)	11 (1.8)
2–3 times/month	37 (19.9)		29 (5.6)	69 (5.6)	254 (22.6)	67 (13.2)	120 (19.0)	16 (2.6)
Once a month	35 (18.8)		15 (2.9)	93 (7.6)	136 (12.1)	50 (9.9)	47 (7.4)	28 (4.6)
Only during school breaks/holidays	37 (19.9)		56 (10.9)	234 (19.1)	133 (11.8)	90 (17.8)	54 (8.6)	43 (7.0)
Not applicable	49 (26.3)		306 (59.5)	756 (61.8)	245 (21.8)	238 (46.9)	249 (39.5)	516 (84.0)
Live in class time								
Parents	30 (61.2)		168 (33.0)	592 (48.8)	205 (18.1)	147 (28.9)	206 (32.8)	83 (13.5)
Alone	12 (24.5)		62 (12.2)	98 (8.1)	132 (11.7)	124 (24.4)	30 (4.8)	174 (28.2)
Boyfriend/girlfriend	3 (6.1)		48 (9.4)	94 (7.7)	107 (9.5)	81 (15.9)	64 (10.2)	185 (30.0)
Colleagues/friends	—		103 (20.2)	243 (20.0)	614 (54.3)	112 (22.0)	289 (45.9)	4 (0.6)
Other family	0		44 (8.6)	63 (5.2)	99 (8.8)	77 (15.1)	38 (6.0)	155 (25.2)
Others	4 (8.2)		84 (16.5)	123 (10.1)	41 (3.6)	6 (1.2)	48 (7.6)	5 (0.8)

To guarantee model linearity, PHQ‐9 and GAD‐7 scores were transformed into the square root of the (score + 1). The selection of fixed effects was made using a backward elimination procedure based on the likelihood ratio test, AIC, and BIC criteria. The likelihood ratio test was used to evaluate the significance of random effects. Residual analysis was performed to evaluate the assumptions of the model, i.e., homogeneity of the level‐1 residuals, normality of the residuals, and the independence between level‐1 and level‐2 residuals. Marginal (*R*_m_^2^) and conditional (*R*_C_^2^) coefficients of determination were used to obtain the explanatory power of the fitted model [28].

Measurement invariance of the PHQ‐9 and GAD‐7 scales across the countries was evaluated using Multigroup Confirmatory Factor Analysis (MGCFA) with a hierarchical procedure (configural, metric, and scalar models), estimated with the Weighted Least Squares Mean and Variance adjusted estimator (WLSMV) for ordinal data. We used ΔCFI ≤ 0.01 and ΔRMSEA ≤ 0.015 as criteria for invariance [29].

Since no information was available about the age of Spanish students, and given the belief that this variable is quite important to include in the model—especially because Swedish students have significantly different age profiles compared to other countries—three modeling approaches were considered: (1) use information from all seven countries without including age variable in the model; (2) exclude Spanish students from the analysis; and (3) impute age for Spanish students based on data from Portugal and France (about age, sex, area of study, study year, study cycle, and working‐student status), using the classification and regression trees (CART) method. CART imputation was chosen because the resulting age distribution did not differ significantly from the original, whereas the predictive mean matching (PMM) method produced a significantly different distribution. The Kolmogorov–Smirnov test was used to compare distributions. Five datasets were generated using multiple imputation with the CART method (mice package, R). Convergence diagnostics indicated stable estimates across iterations. Linear models were then fitted and pooled using Rubin’s rules. The proportion of variance and fraction of missing information suggested only moderate uncertainty, indicating that the imputations were plausible. In the pooled results, sex and course area were not significantly associated with age (*p* > 0.05).

About sex, a residual number of students respond other (*n* = 19), so we imputed this figure to males, as it would not influence the analysis. Note that we did not ask the gender of the participants but rather their sex at birth.

Among the tested models, the least effective fit was observed in the model that excluded age, highlighting the importance of including this variable. The models which either excluded Spanish students or included them with imputed age data produced nearly identical coefficient estimates and model fit statistics. These findings were consistent across both the anxiety (GAD‐7) and depression (PHQ‐9) models. Based on these results, the final model presented includes all countries, with imputed age values for Spanish students (Tables S1 and S2).

Statistical analysis was performed using R software (v. 4.4.0). The package lme4 was used to adjust the LMMs, and the package mice were used to impute missing values. A significance level of 0.05 was used.

### 2.5. Ethical Procedures

This cross‐sectional study was carried out in accordance with the ethical principles of research involving human participants, following international guidelines, such as the Declaration of Helsinki, and the institutional regulations of each of the institutions involved. It received a positive opinion from the ethics committee of each of the institutions involved.

All participants received detailed information about the objectives of the study, the procedures, confidentiality measures, and their right to withdraw at any time without consequences. Digital informed consent was obtained from each participant before the questionnaire was administered, ensuring voluntary and anonymous participation.

Access to the data was restricted to the research team.

## 3. Results

### 3.1. Participants and Sociodemographic Characteristics

A total of 5305 responses were obtained with authorized consent (1136 in Portugal, 633 in Spain, 549 in Ireland, 1392 in Italy, 637 in Sweden, 417 in France, and 541 in Romania). From this sample, 473 questionnaires were eliminated (35 from Ireland, 166 from Italy, 21 from Sweden, 219 from France, and 32 from Romania) because it was not possible to calculate scores on at least one of the MHI‐5, GAD‐7, and PHQ‐9 scales. In addition, two more questionnaires (one from Portugal and one from Spain) were removed as they failed to answer more than half of the remaining questions. Therefore, the final sample consisted of 4830 completed responses.

Most students were female (67.3%–80.7%). Swedish students were the oldest, while Portuguese and French students were the youngest (Table 1).

Most of the Swedish students were in a relationship, living together, while in all other countries, being single was the most common status. The highest percentage of students relocated from the official residence was observed among French and Portuguese students, and the lowest was among Swedish students. The lowest mean age was observed in France (*M* = 20.95 and SD = 2.56), whereas the highest was found in Sweden (*M* = 32.51 and SD = 10.89). The remaining mean ages were as follows: Portugal (*M* = 21.01 and SD = 4.88), Romania (*M* = 22.44 and SD = 4.39), Ireland (*M* = 23.82 and SD = 8.65), and Italy (*M* = 24.25 and SD = 6.26) (Table 1). Additional sociodemographic characteristics can be found in Table 1.

### 3.2. Mental Health

In our study, the proportion of anxiety symptoms (moderate to severe) was 41.58% (95% CI: 40.18%−42.98%) and of depressive symptoms (moderate to severe) was 45.80% (95% CI: 44.39%−47.22%).

The MHI‐5 score significantly differs by country (*F*(6, 1456.7) = 38.039, *p* < 0.001). Swedish and Romanian students presented better mental health, whereas Irish, Italian, and French students present a lower MHI‐5 score (Figure 1a).

Figure 1Score MHI‐5 (a), GAD‐7 (b), and PHQ‐9 (c), by country (different letters represent significant differences with *p* < 0.05).

**Figure:**
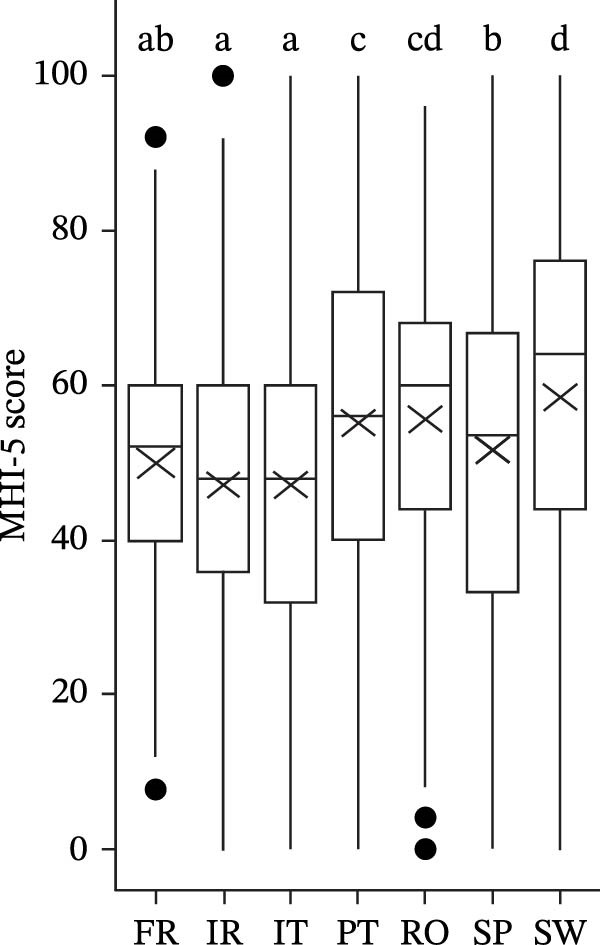
(a)

**Figure:**
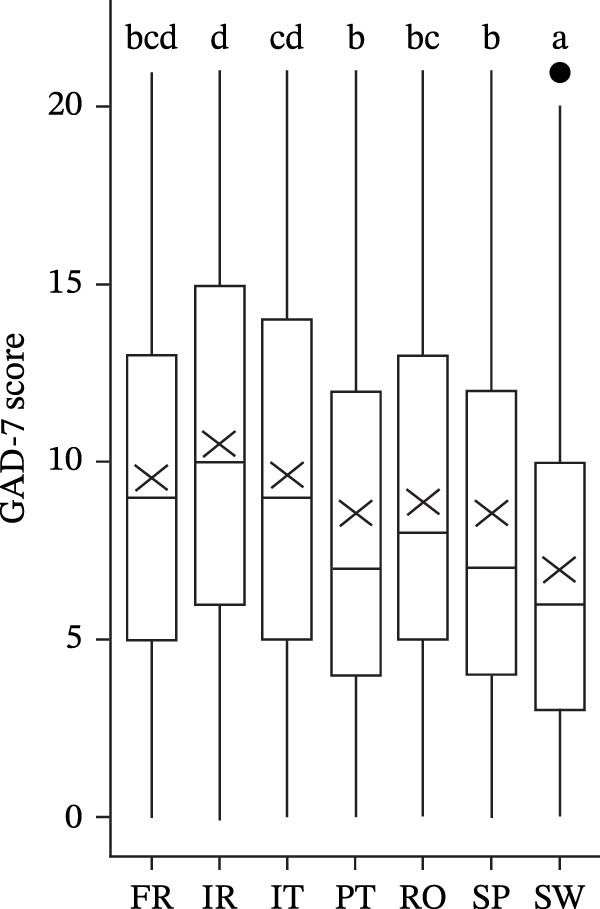
(b)

**Figure:**
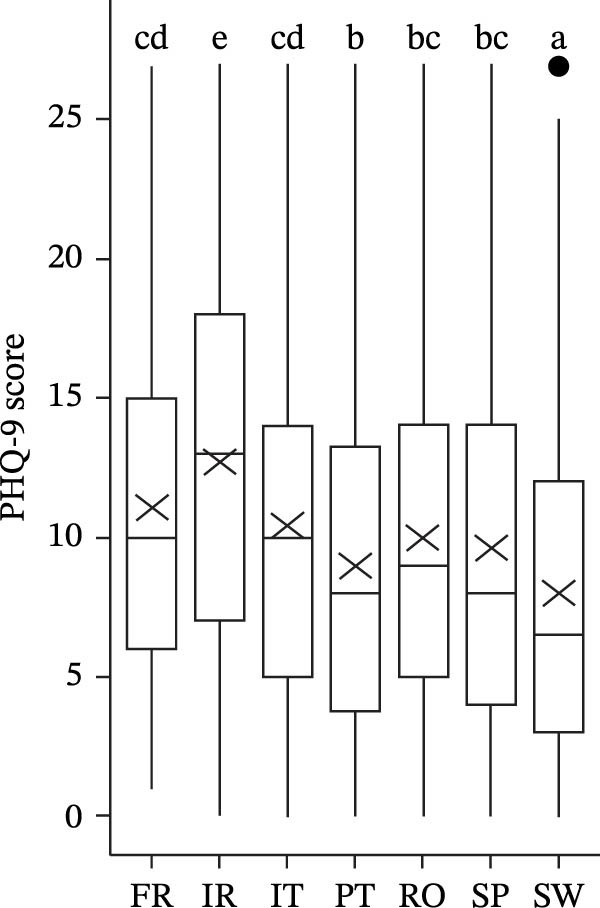
(c)

For anxiety symptoms, the GAD‐7 (transformed as $\sqrt{\text{score}+1}$) significantly differ by country (*F*(6, 1425.7) = 27.654, *p* < 0.001). Swedish students present lower levels of anxiety symptoms, whereas Irish, Italian, and French students present higher GAD‐7 scores (Figure 1b and Table 2).

**Table 2:** Distribution of students (%) by anxiety and depressive symptoms, by country.

Symptoms	France	Ireland	Italy	Portugal	Romania	Spain	Sweden
Anxiety symptoms (GAD‐7)							
Minimal	22.2	18.5	18.2	25.3	24.8	26.2	41.2
Mild	31.8	28.6	34.3	35.6	33.8	36.5	30.8
Moderate	26.8	25.5	26.3	23.6	24.4	22.6	15.7
Severe	19.2	27.4	21.2	15.6	17.1	14.7	12.2
Depressive symptoms (PHQ‐9)							
None‐minimal	22.7	20.2	25.4	36.2	31.8	32.9	42.9
Mild	24.7	16.7	24.3	24.0	21.2	24.8	23.1
Moderate	23.7	22.2	25.9	18.2	23.0	18.2	17.0
Moderately severe	18.7	21.6	14.8	13.7	12.6	13.4	11.9
Severe	10.1	19.3	9.6	7.8	11.4	10.7	5.2

Among Irish students, 27.4% present severe symptoms of anxiety, while 25.5% show moderate symptoms. In contrast, 21.2% of Italian students and 19.2% of French students exhibit severe symptoms, with 26.3% and 26.8%, respectively, experiencing moderate symptoms. Meanwhile, 41.2% of Swedish students report none or only mild symptoms of anxiety (Figure 1b and Table 2).

The depression score PHQ‐9 (transformed as $\sqrt{\text{score}+1}$) significantly differs by country (*F*(6, 1433.8) = 32.960, *p* < 0.001). Swedish students present lower levels of depression symptoms, whereas Irish, Italian, and French students present higher PHQ‐9 scores (Figure 1c and Table 2). Around 19.3% of Irish students present severe symptoms of depression symptoms, while 21.6% exhibit moderately severe symptoms. In contrast, 42.9% of Swedish students and 36.2% of Portuguese students report none or only mild symptoms of depression (Figure 1c and Table 2).

#### 3.2.1. Factors Associated With Depression and Anxiety Symptoms

The model suggests that age (centered), sex, socioeconomic status, academic performance, having a history of previous diagnosis of mental health issues, and taking psychiatric medication are significantly associated with the depressive symptomatology. In addition to these variables, the year of study is significantly associated with anxiety symptoms (Table 3).

**Table 3:** Fitted linear multiple mixed regression models to PHQ‐9 and GAD‐7 scores transformed, with age imputed for Spanish students.

Predictors	PHQ‐9				GAD‐7				
	Estimate	SE	df	*t*	Estimate	SE	df	*t*	*p*‐Value
(Intercept)	3.939	0.123	11.90	32.05	3.388	0.099	17.7	34.25	<0.001
Age (centered)	−0.019	0.002	4467.0	−9.144	−0.017	0.002	4327	−8.92	<0.001
Sex (male/other vs. female)	−0.236	0.031	4514.0	−7.58	−0.295	0.029	4490	−10.16	<0.001
Previous mental diagnosis (yes vs. no)	0.467	0.036	4519.0	13.01	0.388	0.035	4492	11.58	<0.001
Psychiatric medication (yes vs. no)	0.457	0. 036	4514.0	12.27	0.351	0. 039	4490	10.36	<0.001
Socioeconomic level (ref. low)	—	—	—	—	—	—	—	—	<0.001
Medium	−0.321	0.049	4516.0	−6.53	−0.190	0.046	4492	−4.15	<0.001
High	−0.495	0.062	4517.0	−8.01	−0.309	0.058	4493	−5.36	<0.001
Academic performance (ref. poor)	—	—	—	—	—	—	—	—	<0.001
Fair	−0.270	0.062	4514.0	−4.359	−0.165	0.058	4489	−2.86	<0.001
Average	−0.598	0.055	4514.0	−10.820	−0.322	0.052	4900	−6.25	<0.001
Good	−0.857	0.057	4518.0	−15.149	−0.493	0.053	4494	−9.35	<0.001
Excellent	−0.967	0.072	4516.0	−13.399	−0.511	0.067	4493	−7.60	<0.001
Study year (2nd or more vs. 1st year)	—	—	—	—	0.083	0.027	4494	3.11	0.002
**Random effects**									
**Group: parameter**	**Estimate**	**SD** 0.271 (0.158; 0.476)			**Estimate**	**SD** 0.197 (0.113; 0.346) 0.848 (0.830; 0.865)			
Country: (intercept)	0.074				0.039				
*σ* ^2^	0.835	0.914 (0.894; 0.932)			0.720				
*N*_Country_	7	—	—	—	7	—	—	—	—
Adjusted ICC	0.081	—	—	—	0.051	—	—	—	—
$R_{m}^{2}$ and $R_{c}^{2}$	0.225	0.287	—	—	0.167	0.209	—	—	—

*Note: t*, *t* value.

Abbreviations: df, degrees of fit; SD, standard deviation; SE, standard error.

For each additional year of age above the sample mean, the transformed PHQ‐9 score decreases by 0.019 pts and the GAD‐7 by 0.017. Being a female student is associated with a significantly higher score, with an increase of 0.236 pts in depressive symptomatology and 0.295 in symptoms of anxiety compared to male/other. Students with medium and high socioeconomic status show significantly lower depressive symptoms than those with low socioeconomic status, 0.321 and 0.495 pts, respectively, and 0.190 and 0.309 pts in symptoms of anxiety. Depressive symptoms also decrease with higher academic performance, between 0.270 and 0.967 pts when the performance is fair or excellent, respectively, and between 0.165–0.511 pts in the transformed GAD‐7 score. Students with previous mental disorder diagnoses have significantly higher values of depressive symptoms than those without previous mental disorder diagnoses (around 0.467 pts) and similarly for anxiety (around 0.388 pts). Students using psychiatric medication have significantly higher values of depression and anxiety symptomatology than those not using psychiatric medication, around 0.457 and 0.351 pts, respectively. Students in their second year or more years of study have significantly higher values of anxiety than students in the first year (around 0.083 pts).

The models explain a moderate amount of variance in depression (*R*_*m*_^2^ = 22.5% and *R*_*m*_^2^ = 28.7%) and anxiety symptoms (*R*_*m*_^2^ = 16.7% and *R*_*m*_^2^ = 20.9%, Table 3).

For the PHQ‐9, scalar invariance was supported (ΔCFI = −0.001 and ΔRMSEA = 0.013), indicating that mean depression scores can be validly compared across the seven countries. For the GAD‐7, metric invariance was supported (ΔCFI = 0.004 and ΔRMSEA = −0.034)—indicating that the item‐factor relationships are equivalent—but scalar invariance was not achieved (ΔCFI = −0.004 and ΔRMSEA = 0.048). Attempts to obtain partial scalar invariance were not sufficient to meet the ΔRMSEA criterion. Therefore, comparisons of mean anxiety scores across countries should be interpreted with caution.

The ICC showed that about 8% of the variance (for PHQ‐9; and 5% for GAD‐7) was between countries, indicating a modest but meaningful country‐level influence. For both models, the country‐level intercept variance was moderate, indicating differences in average depression scores across countries, and the residual student‐level variance was larger, suggesting that most variability occurs between individuals within countries.

The random effects suggest that France, Ireland, Italy, and Romania have higher PHQ‐9 scores than the baseline, with Ireland and Romania with the highest deviation and France with just a slightly higher deviation than the average. On the other hand, Portugal, Spain, and Sweden have significantly lower scores, after accounting for fixed effects, with Portugal returning lower scores.

For anxiety symptoms, the random effects suggest that the intercept (baseline) for France is in line with the overall sample average. Participants from Ireland, Italy, and Romania reported higher GAD‐7 scores than the baseline, with Italy showing the highest positive deviation from the overall model average. In contrast, Portugal, Spain, and Sweden have significantly lower scores, after accounting for the fixed effects, with Sweden showing lower scores relative to the overall model average. These differences should be viewed with caution and treated as variations in how symptoms are reported, rather than absolute differences in anxiety levels, as the GAD‐7 scores may not be directly comparable across these different cultures.

### 3.3. Help Seeking Behavior

**Figure 2:**
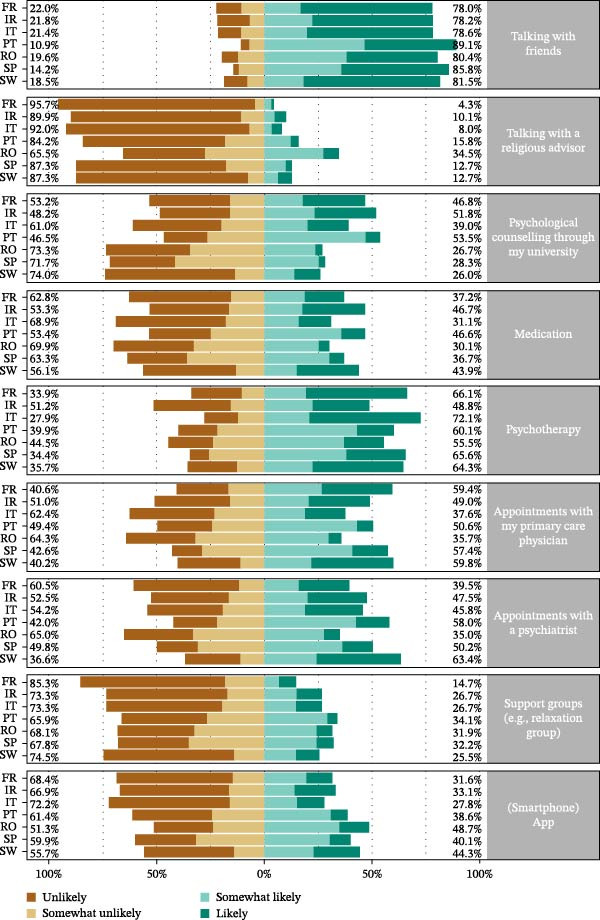
Forms of help by the likelihood of being used by participants who currently have mental distress, or if they develop it in the next year, by country.

In general, by country, the type of support which students are most likely to seek in the event of mental distress is talking with friends or using psychotherapy. The type of support that students are least likely to seek is talking with a religious advisor, participating in support groups, or using smartphone applications (Figure 2).

Significant between‐country differences were observed for all forms of help‐seeking behavior (Figure 2 and Table S3).

Significant differences were found in almost all forms of support by presence vs. absence of anxiety and depression symptoms (Table S4). Talking with friends, talking with a religious advisor, psychological counseling through my university, appointments with my primary care physician, and support groups (e.g., relaxation group) were more mentioned by students without anxiety symptoms as well as by students without depression symptoms. By contrast, medication, psychotherapy, appointments with a psychiatrist, and smartphone app were more frequently mentioned by students with symptoms of anxiety and depression.

### 3.4. Support Seeking Barriers

**Figure 3:**
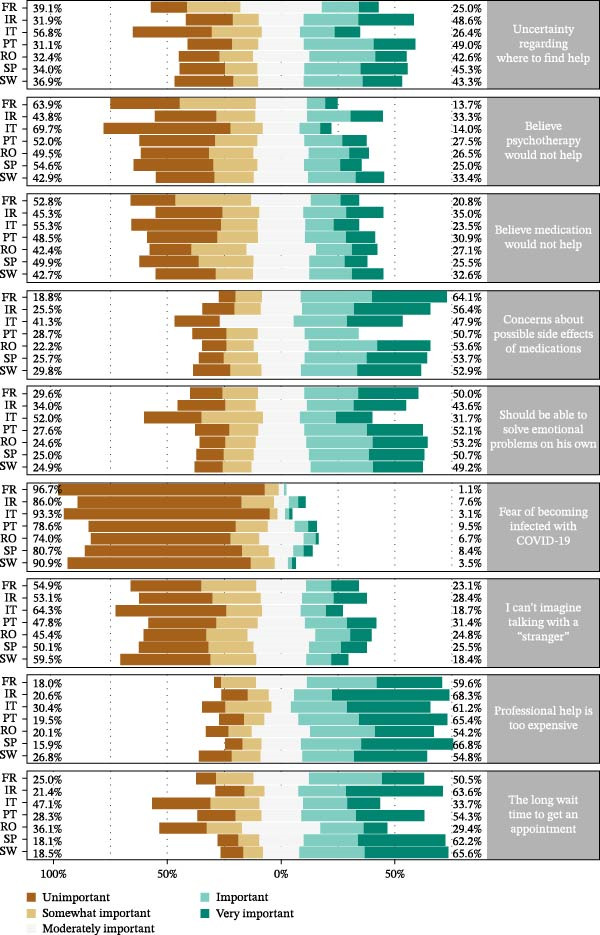
Reasons for participants who currently have mental distress, or if they develop it in the next year, not to seek help, by country.

In general, the barriers to seeking support that students consider most important are professional help is too expensive, the long wait time to get an appointment, concerns about possible side effects of medications, or that they should be able to solve emotional problems on their own (Figure 3).

Significant differences were found between countries in support‐seeking barriers (Figure 3 and Table S5). For example, students from Ireland, Portugal, Spain, and Sweden consider uncertainty regarding where to find help an important barrier, while French and Italian students perceived it as less important. The cost of professional help is seen as a significant barrier by Irish, Spanish, and Portuguese students, whereas Italian, French, Swedish, and Romanian students attach less importance to it.

Significant differences were found in some barriers to seeking help by presence vs. absence of anxiety and depression symptoms (Table S6). Uncertainty regarding where to find help, concerns about possible side effects of medications, should be able to solve emotional problems on his own, I cannot imagine talking with a “stranger,” professional help is too expensive, and the long wait time to get an appointment were more referred by students with anxiety symptoms as well as by student with depression symptoms. On the contrary, fear of becoming infected with COVID‐19 was more mentioned by students without symptoms of depression.

## 4. Discussion

This discussion will reflect on the aims outlined for this study, starting with a discussion of the factors associated with depressive and anxiety symptoms of university students, followed by a reflection of the barriers and facilitators to seeking help.

### 4.1. Factors Associated With Depressive and Anxiety Symptoms

For both anxiety and depressive symptoms, it was found that there was a significant association in the following factors: age, sex, socioeconomic status, perceived academic performance, being diagnosed with a mental disorder, and taking psychiatric medication. Anxiety was associated with the year of the course, although there are differences between countries, the sociodemographic, academic, and clinical characteristics have a greater association on the results.

### 4.2. Sociodemographic Factors

A meta‐analysis with university students about anxiety, which included a total of 83 studies (*N* = 130,090 students), calculated a weighted average prevalence of 39.65% (95% CI: 35.72%–43.58%) for anxiety [30]. In our study, the proportion was 41.58% (95% CI: 40.18%–42.98%).

In the same meta‐analysis, of the studies that analyzed the difference between the sexes, eight studies concluded that being female was associated with a higher anxiety score, and only one study reported higher anxiety scores in men. Furthermore, 19 studies concluded that women were more likely to score above the threshold for anxiety than men. About age, most studies found no significant differences between it and anxiety. However, of those that did find differences, six reported greater anxiety in younger students, and only two found worse results for older students [30].

In this study, the younger the students, the higher the level of anxiety and depression symptoms. Being female was associated with higher levels of anxiety and depression symptoms than being male. More recently, studies carried out in other countries have revealed similar results, with younger students and women showing worse levels of anxiety and/or depression, for example, in Jordan (*N* = 1241) [31], Greece (*N* = 1497) [32], and Saudi Arabia (*N* = 5140) [33]. Furthermore, a study carried out in southern Africa found that the risk of developing any mental disorder was lower among older students than among younger ones (RR = 0.7 and 95% CI = 0.7–0.8) and high among female students (RR = 1.2 and 95% CI = 1.1–1.2) than male [34].

Through this analysis, it can be inferred that the data are consistent in terms of women having more anxiety and depressive symptoms than men. There are several factors that can contribute to this, such as the coping strategies used by men and women, the stressors they are exposed to, the social relationships they establish, and the personal resources and vulnerabilities they develop [35]. However, it is also important to bear in mind that in cultural and social terms, there is still a stigma associated with mental disorders, especially for men [36]. This may be associated with a greater tendency among men in distress to withdraw, to seek less help, and to be underrepresented in questionnaire responses. In fact, in most mental health studies, it is more women than men who respond to.

As for the chronological age of the students, there are more studies reporting that younger people have worse mental health, and the results differ between studies, which indicates that there are other associated factors, and chronological age alone does not fully explain the differences, as were found in this research.

This study shows that students who report having a lower socioeconomic status are the ones with the highest severity of anxious symptoms and/or depressive symptoms. These data are corroborated in studies from other countries, as confirmed by a meta‐analysis that included 64 studies (*N* = 100, 187), which reported that there is an association between low socioeconomic status and worse mental health [5]. A more recent systematic review of the literature, with studies up to the year 2022 (78 studies included), aimed to analyze the association between socioeconomic status and mental disorder in university students, concluded that most studies reported an association between higher symptoms of depression and anxiety and lower socioeconomic status [37]. These data can be associated with access to activities such as physical exercise, recreational activities, greater socialization (for example, more opportunities to carry out activities that involve spending money with friends), and better access to healthcare services [38]. Therefore, it is important that university policies and those countries in general focus on providing the best conditions for families. Socioeconomic status is also a public health issue.

### 4.3. Academic Factors

Students with a perception of poorer academic performance are those who present with more severe levels of anxiety and depressive symptoms. Recent evidence demonstrates similar results [39–41]. This association may be bidirectional. On one hand, poorer mental health is associated with lower academic performance and a perception of lower achievement; for instance, symptoms are linked to increased difficulties in maintaining concentration. Conversely, low academic performance is related to a higher prevalence of anxiety and depressive symptoms, which co‐occur with low self‐esteem, feelings of worthlessness, and uncertainty.

The current study concludes that students in the first year have fewer anxious symptoms than students in the second year or more in their academic studies. Regarding depression, this study did not demonstrate significant results.

A meta‐analysis reported that of 34 studies that studied year of study and anxiety, no consistent relationship was found between these variables. Four of these studies concluded that the earlier the year of study, the lower the anxiety. The same meta‐analysis reported that five studies concluded that first‐year students were more likely to score above the anxiety threshold, and another five studies concluded that later years were more likely to score above the anxiety threshold [30]. Therefore, the data are not consistent across studies and would require more scientific variables to better understand this phenomenon. Although definitive conclusions cannot be derived, we can say that first‐year students may be more anxious because they are experiencing a large change in their lives, which requires adaptation; on the other hand, they are in a situation where everything is novel, and they tend to socialize more which is important for mental health. The course requirements also change, and the approach to the end of the course may also cause more anxiety.

### 4.4. Clinical Factors

In relation to clinical factors, in our study, being diagnosed with a mental disorder and taking psychiatric medication was associated with an increased level of anxiety and depressive symptoms. Similarly, a study carried out in Greece reveals that students who were receiving psychological or psychiatric treatment had severe levels of anxiety and depression [32]. Similar results were observed in a study of Malaysian university students, which found that preexisting medical, depressive, and anxiety disorders were associated with higher depressive and anxiety symptoms after adjustment for age, sex, and marital status [42]. Taking medication was one of the factors associated with increased levels of anxiety in other studies, such as in Jordan [31]. These results merit discussion, as those who have a diagnosis of a disorder and take medication assume that their mental health is affected; however, treatment and medication should lower levels of anxiety and depression. This highlights the need to improve the treatment of mental disorders and to implement psychotherapeutic interventions in addition to medication to help reduce symptoms.

### 4.5. Barriers and Facilitators to Seeking Help

In our study, we found significant differences in the forms of support, namely, that students without anxiety or depressive symptoms utilized strategies such as talking with friends, speaking with a religious advisor, psychological counseling through their university, appointments with a primary care physician, and support groups, such as relaxation groups. In fact, mental health literacy and psychoeducation play a critical role in increasing support‐seeking behaviors. Public awareness campaigns, school‐based mental health programs, and online resources have been shown to improve recognition of symptoms and reduce stigma. Similarly to other studies, a review of facilitators in adolescents found that psychoeducation on mental health issues, in addition to having trusted adults such as teachers and counselors, significantly improved help‐seeking intentions [43].

Social support from family, peers, and community networks is another key facilitator. Individuals who feel supported and encouraged by friends and family are more likely to seek professional help. A study focused on LGBT + university students found that supportive university environments, peer groups, and affirming mental health professionals helped reduce barriers to seeking care [44]. Similarly, in a study from Saudi Arabia, increasing societal and family awareness were identified as crucial factors in improving support‐seeking rates [45].

Alternatively, in this current research, the students with symptoms of anxiety and depression mentioned significant use of medication, psychotherapy, appointments with a psychiatrist, and smartphone apps as facilitators, which constitute more “clinical” strategies. One hypothesis is that these students already have experience with these professionals and have a sense of familiarity with them. A systematic review on self‐harm in young people found that assurance of confidentiality, nonjudgmental support, and prior positive experiences with professionals significantly increased the likelihood of seeking support [46].

Another source of support is the use of online and digital applications, such as online and anonymous mental health resources, which provide another accessible means for individuals to seek support. Many young adults prefer digital platforms, chat services, and teletherapy as these cater to anonymity, reduced stigma, and immediate support. Online mental health services are particularly valuable for those who fear discrimination or lack access to in‐person care [45].

In general, students are more likely to choose facilitators such as talking with friends and attending psychotherapy and are more unlikely to talk with a religious advisor, participate in support groups, or use smartphone apps. Facilitating mental health help‐seeking among students is a global concern, with diverse factors influencing this behavior depending on sociocultural and institutional contexts. However, there appears to be a dearth of literature about specific facilitators related to specific countries. For instance, in Chile, facilitators among LGBT + university students included positive prior experiences with mental health services, which can be related to the findings of this study about attending psychotherapy, as well as the social support from parents, partners, and friends [44]. Contrary to the findings in this research, data from Saudi Arabia identified facilitators for young adults demonstrated online therapy as a key enabler, offering privacy and convenience in a cultural context where stigma and family dynamics often hinder formal help‐seeking [45], which can explain the cultural differences with European countries. In the United Kingdom, the COVID‐19 pandemic paradoxically facilitated support‐seeking among medical students, increased attention to mental health during this period helped reduce stigma, and encouraged students to seek support more openly [47]. A study by Nething et al. [48] in Germany presents the implementation of an intervention program, detailing facilitators that encourage access to mental health services within the context of German higher education institutions. This research included components on peer support and community building, which aimed to reduce isolation and promote collective well‐being [48]. This strategy reinforces the importance of peers and friends as a major facilitator.

Regarding the barriers to seek support, the students of this study which experienced anxiety or depressive symptoms mentioned uncertainty regarding where to find help, concerns about possible side effects of medications, the ability to solve emotional problems on our own, the idea of not being able to imagine talking with a “stranger,” the concept that professional help is too expensive, and the long wait time to get an appointment were prominent factors. Again, many of these barriers have a clinical feature, where autonomy and self‐reliance are additional obstacles, especially among young adults and students. Many individuals prefer to manage their struggles alone rather than rely on external support. Studies on suicide risk in students highlight that self‐reliance and questioning the seriousness of their own needs prevent many from seeking professional help [49]. This tendency is also linked to cultural and family beliefs, particularly in communities where discussing mental health issues is discouraged. In some populations, mental health issues are seen as personal weaknesses, and there is pressure to “deal with problems independently” rather than seeking professional support [43]. Financial and structural barriers are also significant concerns; for example, the expense of mental health services, long waiting times, and lack of access to professionals disproportionately affect individuals from low‐income backgrounds and marginalized communities.

A study on mental health help‐seeking in Saudi Arabia found that financial limitations and the unavailability of culturally sensitive services were major barriers [45]. Similarly, among LGBT + individuals, the high cost and lack of professionals trained in gender and sexuality issues were significant obstacles [44].

Regarding the results per country, the barriers considered more important were the concerns about possible side effects of medication and the duty of being able to solve emotional problems on one’s own. The barriers perceived as less important were the belief that psychotherapy will not help, the fear of becoming affected by COVID‐19, and the idea of not imagining talking to a stranger.

In the study of Noorwali et al. [45], many participants in a qualitative study cited reluctance to discuss personal issues with strangers, low mental health literacy, and a widespread belief that psychological struggles should be handled within the family [45]. It seems that in European countries, the focus is more on individual strategies, centered on one’s (lack of) abilities to face mental health struggles. Although several studies mention the presence of stigma as the main barrier [44, 46, 49], in our findings, the stigma was not clearly mentioned as an important aspect. This may be because in several European countries, mental health has been a subject of discussions and workshops in academic environments, as well as in social media. Nevertheless, Nething et al. [48] mentioned that public stigma involves societal attitudes that label individuals with mental illness as lazy, disorganized, or less competent, and when individuals internalize these societal judgments, they develop self‐stigma, which is associated to shame, reduced self‐worth, and, ultimately, avoidance of help‐seeking behavior. This conceptualization of self‐stigma can be related to the sense of duty that each individual should solve the emotional problems by himself [48]. This conceptualization of self‐stigma can be related to the sense of duty that each individual should solve the emotional problems by himself.

While this study identified significant variations in anxiety and depressive symptoms among students from different countries, these cross‐national differences should be interpreted cautiously. Structural and cultural factors, such as the availability and accessibility of mental health services, national educational policies, social welfare systems, and cultural attitudes towards mental health, likely influence how students experience and report symptoms, as well as their help‐seeking behaviors. For example, countries with extensive student support services and mental health literacy programs may mitigate the impact of socioeconomic stressors, whereas in contexts with limited resources or higher stigma, students may experience increased vulnerability. Future research should explicitly consider these contextual factors to better understand international differences and to inform culturally sensitive interventions.

## 5. Strengths and Limitations

This study covered seven European countries (Portugal, France, Italy, Spain, Sweden, Ireland, and Romania) and involved 4830 higher education students, allowing for comparisons of cultural differences and greater robustness in the results obtained. LMMs were considered in the statistical analysis, making it possible to control for variations between the different contexts and to test various variables associated with depressive and anxiety symptoms. In addition to exploring depressive and anxious symptomatology, this study identified (with statistically significant results) the forms of help that students are most likely to use and prefer, as well as the barriers they feel when seeking help in the event of mental distress. These results provide a comprehensive view that can contribute to a more effective approach to promoting the mental health of higher education students.

Some limitations may influence the results obtained, such as the cross‐sectional design, with data collected at a single time point and by convenience. This approach restricts the ability to draw causal inferences and to capture potential changes in key variables over time. To address these limitations, future phases of the study will adopt a longitudinal design, enabling the tracking of individual trajectories and temporal dynamics. The use of repeated measures will provide more robust evidence regarding the temporal sequencing of events and strengthen the validity of causal interpretations.

The study used convenience sampling through online surveys, which may have introduced selection bias and limited the generalizability of the findings. Participants who chose to participate may differ from nonrespondents, for example, in terms of mental health awareness or engagement, potentially leading to an overrepresentation of students with higher symptom reporting or greater interest in mental health topics. While this cannot be fully mitigated, it should be taken into account when interpreting the results.

The sample was predominantly female (67.3%–80.7% across countries), which may have influenced the results, as female students generally report higher levels of mental health symptoms. This gender imbalance could lead to an overestimation of overall prevalence and limit the generalizability of the findings to the broader student population.

A further potential limitation stems from the unavailability of several key variables that might act as important predictors or confounding variables. These include, but are not limited to, physical illnesses, lifetime trauma, and substance use.

The low reliability found in the MHI‐5 of the French subsample represents a limitation of the study. Although this measure was used descriptively, the low reliability may affect the characterization of this subgroup, and results should therefore be interpreted with caution.

The measurement instruments are self‐report, which could lead to a bias in social desirability that depends on the subjective perception of the students. There was a lack of data for variables such as the age of Spanish students, and data imputation was used, which despite the use of robust statistical methods could lead to some inaccuracy in the results of the multivariate analysis.

The GAD‐7 did not achieve scalar invariance across countries, meaning that mean anxiety scores among countries may not be directly comparable, and cross‐country differences should be interpreted cautiously; item intercepts suggest that cultural nuances in reporting may influence results. By contrast, scalar invariance was supported for the PHQ‐9, allowing valid comparisons of depression scores across countries.

## 6. Conclusion

The study showed statistically significant variations in the prevalence and severity of depressive and anxious symptoms among students from the countries under study. In general, Swedish students had lower levels of this type of symptomatology when compared to students from the other countries. Social factors such as socioeconomic status and individual factors such as age, sex, academic performance, medical history of mental disorder, or year of study were significantly associated with depressive and anxious symptoms.

Regarding strategies for seeking help, students preferred talking to friends or accessing psychotherapy. However, barriers such as cost, long waiting times, uncertainty about where to seek help, and the belief that one should solve problems independently were frequently reported. Students’ views need to be considered when reviewing or creating strategies to promote their mental health, considering their individual and cultural needs.

Based on these findings, actionable policy recommendations include the following:

1. Scaled‐up mental health services: Provide different levels of support depending on severity, from peer support and group support to individual psychotherapy.
2. Sliding‐scale fees or subsidized access: Reduce financial barriers to professional mental health support.
3. Integration of mental health modules into curricula: Offer mandatory or elective courses/workshops that teach coping strategies, stress management, and mental health literacy.
4. Streamlined access and reduced waiting times: Improve scheduling systems and increase the availability of services to ensure timely support.
5. Antistigma initiatives: Implement campaigns and educational programs to reduce stigma and self‐stigma related to seeking mental health care, tailored to cultural and individual needs.

These steps provide concrete, evidence‐based strategies to improve mental health support for university students, enhancing accessibility, inclusivity, and overall well‐being.

## Funding

The work is funded by national funds through FCT – Fundação para a Ciência e Tecnologia, I.P., in the framework of the CHRC UID/06291/2025—https://doi.org/10.54499/UID/06291/2025.

## Ethics Statement

This study received a positive opinion from the ethics committee of each of the institutions involved: University of Évora (Ethical Committee from Universidade de Evora, Reference Number 22055, May 4, 2022), University of Gavle (Swedish Ethical Review Authority, Reference Number 2022‐04690‐01, November 2022), University of Parma (Research Ethics Board of the University of Parma, Reference Number 94‐2023‐N, October 25, 2023), University of Oradea (Research Ethics Committee University of Oradea, Reference Number BPPCDI/1061/30.10.2023, October 30, 2023), ATU (Reference Number RSC_ATU_EF_113), University of Angers (Comité d’éthique Université Angers, Reference Number 20240212, February 12, 2024), and University of Extremadura: (La Comisión de Bioética y Bioseguridad de la Universidade de Extremadura, Reference Number 129//2023, September 28, 2023).

## Conflicts of Interest

The authors declare no conflicts of interest.

## General Statement

This research project focused on three areas: (1) mental health (this study), (2) sexual and moral harassment, and (3) substance abuse, such as alcohol, tobacco, and medication. The last two will be the subject of another manuscript. The data from this study will be used for these three publications, although they will not be repeated, as each manuscript has its own specific and distinct objective.

## Supporting Information

Additional supporting information can be found online in the Supporting Information section.

## Supporting information


**Supporting Information** Due to the large number of analyses carried out to process the data, we have sent six tables as Supporting Information to the manuscript, which are duly referenced throughout the manuscript. This is the tables in Supporting Information: Table S1: Comparison of the three models estimated for PHQ9 score. Table S2: Comparison of the three models estimated for GAD‐7 score. Table S3: Kruskal–Wallis and Dunn’s test results for comparison of countries in each form of help. Table S4: Mann–Whitney Wilcoxon test results to check for differences in each form of help by presence of anxiety and depression. Table S5: Kruskal–Wallis and Dunn’s tests results for comparison of countries in each help‐seeking barrier. Table S6: Mann–Whitney Wilcoxon test results to check for differences in each barrier to seek help by the presence of anxiety and depression.

## Data Availability

The data that support the findings of this study are available on request from the corresponding author. The data are not publicly available due to privacy or ethical restrictions.
