# Supplementary material for: Factors Associated With Depressive and Anxiety Symptoms, Barriers, and Facilitators for Seeking Support Among European University Students: A Cross‐Sectional Multicenter Study
Source: Depress Anxiety. 2026 Apr 10;2026:8994187. doi: 10.1155/da/8994187 (PMC13069185; doi:10.1155/da/8994187)
Supplement: Supplementary file 1 — Supporting Information Due to the large number of analyses carried out to process the data, we have sent six tables as Supporting Information to the manuscript, which are duly referenced throughout the manuscript. This is the tables in Supporting Information: Table S1: Comparison of the three models estimated for PHQ9 score. Table S2: Comparison of the three models estimated for GAD‐7 score. Table S3: Kruskal–Wallis and Dunn’s test results for comparison of countries in each form of help. Table S4: Mann–Whitney Wilcoxon test results to check for differences in each form of help by presence of anxiety and depression. Table S5: Kruskal–Wallis and Dunn’s tests results for comparison of countries in each help‐seeking barrier. Table S6: Mann–Whitney Wilcoxon test results to check for differences in each barrier to seek help by the presence of anxiety and depression. [file DA-2026-8994187-s001.docx]

# Supplementary Table 1 - Comparison of the three models estimated for PHQ9 score

# Supplementary Table 2 - Comparison of the three models estimated for GAD-7 score

# Supplementary Table 3 - Kruskal-Wallis and Dunn’s tests results for comparison of countries in each form of help

# Supplementary Table 4 - Mann-Whitney Wilcoxon test results to check for differences in each form of help by presence of anxiety and depression

Supplementary Table 5 - Kruskal-Wallis and Dunn’s tests results for comparison of countries in each help-seeking barrier

# Supplementary Table 6 - Mann-Whitney Wilcoxon test results to check for differences in each barrier to seek help by presence of anxiety and depression

# Supplementary Table 1 - Comparison of the three models estimated for PHQ9 score

| **Predictors** | **Approach 1**  **(excluding Age)** | | **Approach 2**  **(excluding Spain)** | | **Approach 3**  **(age imputation)** | |
| --- | --- | --- | --- | --- | --- | --- |
|  | **Estimate** | **SE** | **Estimate** | **SE** | **Estimate** | **SE** |
| (Intercept) | 3.948 | 0.127 | 3.983 | 0.136 | 3.939 | 0.123 |
| Age (centered) | - | - | -0.019 | 0.002 | -0.019 | 0.002 |
| Sex (Female vs. Male/Other) | -0.257 | 0.032 | -0.250 | 0.033 | -0.236 | 0.031 |
| Previous mental diagnosis (No vs. Yes) | 0.452 | 0.037 | 0.471 | 0.038 | 0.467 | 0.036 |
| Psychiatric medication (No vs. Yes) | 0.430 | 0.037 | 0.429 | 0.039 | 0.457 | 0. 036 |
| Socio economic level (ref. Low) |  |  |  |  |  |  |
| Medium | -0.320 | 0.049 | -0.319 | 0.052 | -0.321 | 0.049 |
| High | -0.515 | 0.063 | -0.498 | 0.065 | -0.495 | 0.062 |
| Academic performance (ref. Poor) |  |  |  |  |  |  |
| Fair | -0.281 | 0.063 | -0.289 | 0.068 | -0.270 | 0.062 |
| Average | -0.596 | 0.056 | -0.602 | 0.061 | -0.598 | 0.055 |
| Good | -0.858 | 0.058 | -0.871 | 0.062 | -0.857 | 0.057 |
| Excellent | -0.980 | 0.073 | -0.993 | 0.077 | -0.967 | 0.072 |
| Emotional relationship (Single vs. Not single) | 0.074 | 0.028 |  |  |  |  |
| Bachelor/Undergraduate (Yes vs. No) | -0.086 | 0.033 |  |  |  |  |
| **Random effects** |  |  |  |  |  |  |
| **Group: Parameter** | **Estimate** | **SD** | **Estimate** | **SD** | **Estimate** | **SD** |
| Country: (Intercept) | 0.078 | 0.279 | 0.079 | 0.282 | 0.074 | 0.271 |
| σ^2^ | 0.848 | 0.921 | 0.827 | 0.909 | 0.835 | 0.914 |
| *N*_Country_ | 7 |  | 6 |  | 7 |  |
| $R_{m}^{2}$ and $R_{c}^{2}$ | 0.207 | 0.273 | 0.228 | 0.296 | 0.225 | 0.287 |

**Note:** SE: Standard Error, SD: Standard Deviation

# Supplementary Table 2 - Comparison of the three models estimated for GAD-7 score

|  | **Approach 1**  **(excluding Age)** | | | **Approach 2**  **(excluding Spain)** | | | **Approach 3**  **(age imputation)** | | |
| --- | --- | --- | --- | --- | --- | --- | --- | --- | --- |
| **Predictors** | **Estimate** | **SE** | **Estimate** | | **SE** | **Estimate** | | **SE** |  |
| (Intercept) | 3.426 | 0.108 | 3.481 | | 0.107 | 3.388 | | 0.099 |  |
| Age (centered) |  |  | -0.018 | | 0.002 | -0.017 | | 0.002 |  |
| Sex (Female vs. Male/Other) | -0.311 | 0.029 | -0.356 | | 0.035 | -0.295 | | 0.029 |  |
| Previous mental diagnosis (No vs. Yes) | 0.379 | 0.034 | 0.393 | | 0.039 | 0.388 | | 0.035 |  |
| Psychiatric medication (No vs. Yes) | 0.343 | 0.034 | 0.332 | | 0.037 | 0.351 | | 0. 039 |  |
| Socio economic level (ref. Low) |  |  |  | |  |  | |  |  |
| Medium | -0.198 | 0.046 | -0.179 | | 0.048 | -0.190 | | -0.190 |  |
| High | -0.333 | 0.058 | -0.320 | | 0.061 | -0.309 | | -0.309 |  |
| Academic performance (ref. Poor) |  |  |  | |  |  | |  |  |
| Fair | -0.164 | 0.058 | -0.180 | | 0.064 | -0.165 | | -0.165 |  |
| Average | -0.302 | 0.052 | -0.334 | | 0.571 | -0.322 | | -0.322 |  |
| Good | -0.504 | 0.054 | -0.501 | | 0.058 | -0.493 | | -0.493 |  |
| Excellent | -0.543 | 0.068 | -0.512 | | 0.073 | -0.511 | | -0.511 |  |
| Study year (2^nd^ or more vs. 1^st^ year) | 0.063 | 0.027 |  | |  | 0.083 | | 0.027 |  |
| Sex (Male/Other) * Previous mental diagnosis (Yes) |  |  | 0.208 | | 0.076 |  | |  |  |
| **Random effects** |  |  |  | |  |  | |  |  |
| **Group: Parameter** | **Estimate** | **SD** | **Estimate** | | **SD** | **Estimate** | | **SD** |  |
| Country: (Intercept) | 0.051 | 0.226 | 0.041 | | 0.202 | 0.039 | | 0.197 |  |
| σ^2^ | 0.733 | 0.856 | 0.726 | | 0.852 | 0.720 | | 0.848 |  |
| *N*_Country_ | 7 |  | 6 | |  | 7 | |  |  |
| $R_{m}^{2}$ and $R_{c}^{2}$ | 0.150 | 0.205 | 0.169 | | 0.213 | 0.167 | | 0.209 |  |

**Note:** SE: Standard Error, SD: Standard Deviation)

# Supplementary Table 3 - Kruskal-Wallis and Dunn’s tests results for comparison of countries in each form of help (The lowest the letter the higher is the likely of using the form of help.

# Countries sharing the same letter do not significantly differ by likelihood of using the form of help at α=0.05).

| Form of help | $\boldsymbol{\chi}_{\boldsymbol{6}}^{\boldsymbol{2}}$ | P value | n | $\boldsymbol{\eta}^{\boldsymbol{2}}$ | FR | IR | IT | PT | RO | SP | SW |
| --- | --- | --- | --- | --- | --- | --- | --- | --- | --- | --- | --- |
| Talking with friends | 44.220 | <0.001 | 4681 | 0.008 | ab | abc | a | bc | c | ab | a |
| Talking with a religious advisor | 431.772 | <0.001 | 4623 | 0.092 | d | c | cd | b | a | b | c |
| Psychological counselling through my university | 234.300 | <0.001 | 4634 | 0.049 | ab | a | bc | a | d | cd | e |
| Medication | 103.005 | <0.001 | 4629 | 0.021 | bcd | a | d | a | cd | abc | ab |
| Psychotherapy | 240.242 | <0.001 | 4641 | 0.051 | ab | c | a | c | c | b | b |
| Appointments with my primary care physician | 164.036 | <0.001 | 4632 | 0.034 | a | b | c | b | c | a | a |
| Appointments with a psychiatrist | 118.454 | <0.001 | 4629 | 0.024 | de | bcd | cd | b | e | bc | a |
| Support groups (e.g., relaxation group) | 122.823 | <0.001 | 4615 | 0.025 | c | b | b | a | a | a | bc |
| (Smartphone) App | 137.909 | <0.001 | 4628 | 0.029 | cd | c | d | bc | a | ab | ab |
| Online intervention | 103.370 | <0.001 | 4574 | 0.021 | d | d | c | a | ab | abc | bc |

# Supplementary Table 4 - Mann-Whitney Wilcoxon test results to check for differences in each form of help by presence of anxiety and depression.

|  | Anxiety | | Depression | |
| --- | --- | --- | --- | --- |
| Form of help | **W** | **P value** | **W** | **P value** |
| Talking with friends | 2908005 | <0.001 | 3051640 | <0.001 |
| Talking with a religious advisor | 2767400 | <0.001 | 2889173 | <0.001 |
| Psychological counselling through my university | 2724376 | 0.020 | 2830321 | <0.001 |
| Medication | 2183523 | <0.001 | 2224850 | <0.001 |
| Psychotherapy | 2435547 | <0.001 | 2561435 | 0.007 |
| Appointments with my primary care physician | 2800451 | <0.001 | 2909440 | <0.001 |
| Appointments with a psychiatrist | 2510631 | 0.012 | 2549332 | 0.007 |
| Support groups (e.g., relaxation group) | 2807066 | <0.001 | 2849812 | <0.001 |
| (Smartphone) App | 2509520 | 0.012 | 2580152 | 0.044 |
| Online intervention | 2527223 | 0.421 | 2644134 | 0.370 |

# Supplementary Table 5 - Kruskal-Wallis and Dunn’s tests results for comparison of countries in each help-seeking barrier (The lowest the letter the higher is the importance assigned to the barrier. Countries sharing the same letter do not significantly differ by importance assigned to the barrier at α=0.05).

| Help-seeking barrier | $\boldsymbol{\chi}_{\boldsymbol{6}}^{\boldsymbol{2}}$ | P value | n | $\boldsymbol{\eta}^{\boldsymbol{2}}$ | FR | IR | IT | PT | RO | SP | SW |
| --- | --- | --- | --- | --- | --- | --- | --- | --- | --- | --- | --- |
| Uncertainty regarding where to find help | 191.181 | <0.001 | 4559 | 0.041 | bc | a | c | a | ab | a | ab |
| Believe psychotherapy would not help | 251.519 | <0.001 | 4533 | 0.054 | b | a | c | b | ab | b | a |
| Believe medication would not help | 54.438 | <0.001 | 4525 | 0.011 | ab | a | b | a | a | a | a |
| Concerns about possible side effects of medications | 47.180 | <0.001 | 4528 | 0.009 | a | ab | c | b | ab | ab | ab |
| Should be able to solve emotional problems on his own | 181.047 | <0.001 | 4543 | 0.039 | bc | c | d | a | a | a | bc |
| Fear of becoming infected with COVID-19 | 286.917 | <0.001 | 4523 | 0.062 | e | c | e | ab | a | bc | d |
| I can’t imagine talking with a “stranger” | 124.490 | <0.001 | 4529 | 0.026 | ab | a | c | a | a | a | bc |
| Professional help is too expensive | 62.739 | <0.001 | 4539 | 0.013 | bcd | a | bc | abc | d | ab | d |
| The long wait time to get an appointment | 401.031 | <0.001 | 4539 | 0.087 | b | a | c | b | c | a | a |

# Supplementary Table 6 - Mann-Whitney Wilcoxon test results to check for differences in each barrier to seek help by presence of anxiety and depression.

| Barrier to seek help | Anxiety | | Depression | |
| --- | --- | --- | --- | --- |
|  | **W** | **P value** | **W** | **P value** |
| Uncertainty regarding where to find help | 2360064 | <0.001 | 2364130 | <0.001 |
| Believe psychotherapy would not help | 2492818 | 0.584 | 2483705 | 0.069 |
| Believe medication would not help | 2530127 | 0.566 | 2553876 | 0.950 |
| Concerns about possible side effects of medications | 2389654 | 0.005 | 2456334 | 0.022 |
| Should be able to solve emotional problems on his own | 2338941 | <0.001 | 2351234 | <0.001 |
| Fear of becoming infected with COVID-19 | 2543673 | 0.219 | 2678738 | <0.001 |
| I can’t imagine talking with a “stranger” | 2421060 | 0.034 | 2388858 | <0.001 |
| Professional help is too expensive | 1956972 | <0.001 | 1951033 | <0.001 |
| The long wait time to get an appointment | 2196569 | <0.001 | 2232510 | <0.001 |
